# SARS-CoV-2 (COVID-19) Vaccine Intentions in Kentucky

**DOI:** 10.13023/jah.0402.04

**Published:** 2022-07-01

**Authors:** Kevin A. Pearce, Emily Messerli, Mary E. Lacy, Brittany L. Smalls, Diane B. Francis, Sukruthi Yerramreddy, Marc Kiviniemi

**Affiliations:** University of Kentucky, kevin.pearce@uky.edu; Kentucky Department for Public Health; University of Kentucky; University of Kentucky

**Keywords:** Appalachia, vaccine hesitancy, COVID-19

## Abstract

**Background:**

At the time of our writing, the COVID-19 pandemic continues to cause significant disruption to daily lives. In Kentucky, the burdens from this disease are higher, and vaccination rates for COVID-19 are lower, in comparison to the U.S. as a whole. Understanding vaccine intentions across key subpopulations is critical to increasing vaccination rates.

**Purpose:**

This study explores COVID-19 vaccine intentions in Kentucky across demographic subpopulations and also investigates the influences on vaccine intention of attitudes and beliefs about COVID-19.

**Methods:**

A population-based survey of 1,459 Kentucky adults was conducted between January 26 and March 20, 2021, with over-sampling of black/African American and Latino/a residents, using online and telephonic modalities. Descriptive statistics characterize the sample and overall vaccine intentions and beliefs. Multivariable linear regression models probed relationships between demographics and vaccination intentions, as well as relationships between vaccination beliefs and vaccination intention.

**Results:**

Of the 1,299 unvaccinated respondents, 53% reported intent to get vaccinated, 16% had not decided, and 31% felt they would not get vaccinated. Lower vaccination intention was independently associated with age, lower educational attainment, black/African American race, lower income, Republican political affiliation, rural residence, and several beliefs: low vaccine safety, low vaccine efficacy, the rapidity of vaccine development, and mistrust of vaccine producers.

**Implications:**

Increasing COVID-19 vaccination rates will help end this pandemic. Findings from this study can be used to tailor information campaigns aimed at helping individuals make informed decisions about COVID-19 vaccination.

## INTRODUCTION

As of spring 2022, the COVID-19 pandemic remains uncontrolled in the U.S., despite social distancing, masking, and the widespread distribution of vaccines against the causative organism, severe acute respiratory syndrome coronavirus 2 (SARS-CoV-2).^1^ The Pfizer COVID-19 vaccine and the Moderna vaccine received FDA Emergency Use Authorization (EUA) in December 2020, with FDA EUA of the Johnson & Johnson COVID-19 vaccine following on February 27, 2021. Insufficient vaccination rates and the emergence of more virulent variants of this virus have rendered the goal of achieving herd immunity in the U.S. highly elusive. For the first few months of 2021, COVID-19 vaccine *access* appeared to be the main determinant of vaccination rates within communities, as the demand for vaccines commonly outstripped the supplies of them. But by April 2021, vaccine *hesitancy* appeared to be dampening vaccination rates, as the demand for COVID-19 vaccination fell significantly across the U.S., even though only 45% of people in the country had received at least one dose of vaccine, and just 32% were fully vaccinated.^2^,^3^ Similarly, in Kentucky, vaccination rates were 41% and 32%, respectively, in April 2021.^2^ Vaccine supplies began to rise significantly beyond demand, even as many Kentucky counties were falling behind the nation in their COVID-19 vaccination rates. As of May 23, 2022, the COVID-19 vaccination population rates in Kentucky were 66% for at least one dose, and 57% for those fully vaccinated, defined as having received two doses of the Pfizer or Moderna vaccine, or one dose of the Johnson & Johnson vaccine.^2^

Vaccine hesitancy is defined as delay in acceptance or refusal of vaccination despite availability of vaccination services.^4^ It is the most likely cause of the reduced demand for COVID-19 vaccination and the looming failure of many communities to reach vaccination rates that could plausibly lead to herd immunity. Vaccine hesitancy (and confidence, the converse of hesitancy) has been widely studied.^4^–^7^ Varying conceptual models have emerged, and the variability of robust drivers of vaccine hesitancy specific to different vaccines and circumstances has been recognized. A unique example in the case of COVID-19 vaccines is the rapidity of their development. A large systematic review of COVID-19 vaccine confidence found that measurable vaccine hesitancy was universal across the U.S. It also found several consistent perceptual determinants of COVID-19 vaccine hesitancy: disease severity, infection risk, vaccine safety, vaccine effectiveness, and vaccine necessity.^8^ Common facilitators of COVID-19 vaccine confidence were influenza vaccine acceptance, trust in government, and the recommendation of doctors.^8^ An assessment of vaccine confidence in the U.S. by the National Vaccine Advisory Committee found similar associations, and also noted that social norms and religious beliefs affect some of these.^7^

Published papers have demonstrated COVID-19 vaccine hesitancy rates (usually the sum of uncertainty and expressed intent to avoid vaccination), or, conversely, rates of positive intention for COVID-19 vaccination.^3^,^8^,^9^ These papers have shown average COVID-19 vaccine hesitancy rates of 28% to 52%, with variance across geographic region, demographic variables, and survey administration times.^3^,^8^ Similar published data and information specific to Kentucky—especially to Appalachian communities—are sparse.

The main goals of this study were to understand COVID-19 vaccine intentions in Kentucky across different demographic subpopulations and to explore the influences of Kentuckians’ attitudes and beliefs about COVID-19 on intention to accept COVID-19 vaccination.

## METHODS

### Participants/Sampling

Adult residents of the Commonwealth of Kentucky comprised the study population. Participants from the survey were primarily drawn from two distinct sources: an online survey panel provided by a local survey research firm (IQS Research) and telephone interviews directed to mobile phone and landline numbers. An online survey was provided to all participants recruited via the online panel and was provided as an option for participants recruited by phone. Additional survey responses were collected via telephone interviews. Surveys were made available in both English and Spanish for respondents. Data collection was conducted from January 26 to March 20, 2021. All results reported here control for survey mode.

Panel surveys are a well-established research method in the social sciences. Panel participants are recruited through various outreach methods—including the use of social media and online targeting—and they agree to answer periodic surveys. Respondents in this study received a modest incentive for their participation in the form of points that could be accumulated and redeemed for small gifts. The response quality of these individuals was regularly monitored. Typical quality measures implemented by panel companies include IP cross-matching to prevent multiple submissions and checks for speeding, habituation, and other quality elements. Companies also employ multiple data quality review scripts and will remove any responses that do not meet the quality standards. In this case, all data were quality checked, but only minimal removals were needed and no more so than on other similar projects.

An additional group of participants were recruited using newspaper, social media, Snapchat, and radio advertisements targeted to the Latino/a community. This outreach was conducted fully in Spanish and was done in partnership with a media outlet that is well known in the Latino/a community. These responses were also quality checked, as described before. This group received no incentive.

Respondents were requested to freely participate in the survey without inducement (beyond the survey panel points described above), and all questions after the screening process were optional. Standard protocols for privacy and personal harm, as they apply to social research, were followed. Respondent identification was anonymous during the analysis process. Demographic information such as gender, income, race, and other factors was provided by the respondents. In addition, paradata (e.g., survey length and mode) and metadata (e.g., IP address and source) were gathered at various stages of the research process. However, identifying information, including phone numbers, was removed and replaced with a respondent ID prior to analysis.

A total of 1,459 adults completed the survey. To allow for stable subpopulation estimates by race and ethnicity, power analyses were conducted to determine the needed sample size of white, black/African American and Latino/a adults. Given the sample size versus their proportions in the population of the state of Kentucky, purposive oversampling of black/African American and Latino/a adults was an *a priori* design feature. This resulted in n=315 black/African American and n=263 Latino/a adults, respectively.

### Measures

The study assessed COVID-19 vaccine intentions, perceptions, attitudes, and beliefs. Survey items were developed by the research team, in consultation with the Kentucky Department for Public Health and the Kentucky Governor’s Office.

#### Rurality and Appalachian Residence

The rurality and Appalachian status of the respondents’ county of residence was assessed. Participants reported county of residence, and the USDA Rural–Urban Continuum Codes (RUCC) were used to classify the relative rurality of it. RUCC provides a nine-category classification in which higher numbers indicate greater rurality.^10^

That report was then used to assign a rurality score for each participant based on the RUCC code for their county of residence. Some RUCC categories were combined because of very small cell sizes (RUCC categories were 1, 2–3, 4–6, 7, and 8–9). Appalachian counties were designated according to criteria used by the Appalachian Regional Commission.^11^

#### COVID-19 Vaccination Beliefs

Beliefs about the COVID-19 vaccine were assessed using nine items from the Carolina HPV immunization attitudes and beliefs scale, modified to have COVID-19 as the vaccination referent.^12^ The scale is a validated, widely used measure of attitudes and beliefs about vaccination. The full set of items is provided in Table 4 and included statements such as, “The COVID-19 vaccine might cause lasting health problems.” Participants indicated their degree of agreement with each item using a four-point, Likert-type response scale with response options of “strongly disagree,” “disagree,” “agree,” and “strongly agree.”

In this report, *intentions to vaccinate* are COVID-19 vaccine specific and reflect whether a given individual plans to receive the COVID-19 vaccine when it becomes available to the individual. Conversely, *general vaccination hesitanc*y is not specific to COVID-19 vaccination; it reflects a general attitude or disposition to avoid or delay vaccinations due to safety or efficacy concerns. One item assessed vaccine hesitancy based on whether an individual reported having ever refused or postponed a vaccination (for the individual or a child) because of concerns about safety or efficacy. This item was developed by Rey et al., based on the standard World Health Organization definition of vaccine hesitancy. Participants responded using categorical response options of “yes,” “no,” or “don’t know.”^13^

#### Vaccination Intentions

Two items assessed vaccine intentions (and were asked in different parts of the survey). The items and response options are provided in Table 2. The mean of the two items was used as the index of intentions to receive the COVID-19 vaccination.

#### Demographics

The following demographic data were collected: age, gender, ZIP Code, education, ethnicity, race, income, health insurance, whether the respondent had a regular healthcare provider apart from mental health, political affiliation, and employment status. All data were self-reported. When analyzing the effects of race and ethnicity on COVID-19 vaccine intentions, three strata were defined: black/African American, Latino/a, and non-Latino/a white. Because there is substantial overlap between the different sociodemographic indicators (e.g., the study found that more than 80% of black/African Americans are also urban residents, and reported income is lower for rural versus urban respondents), all reported differences in intentions based on sociodemographic factors are from analyses that account for the other sociodemographic factors.

### Analyses

All analyses were conducted using STATA 16 (StataCorp., College Station TX)^14^ and used STATA’s complex survey analysis techniques to incorporate survey weights^15^,^16^ so as to provide estimates representative of the adult population of Kentucky. All descriptive and inferential statistics reported here are weighted. The contracted local research firm derived survey weights based on age, race/ethnicity, and gender for each survey respondent. Raw demographics data are also reported.

Descriptive statistics were used to characterize the population and overall vaccine intentions and beliefs. To examine the relation between demography and vaccination intentions (continuous measure), a multivariable linear regression model was estimated with vaccination intentions as the continuous outcome measure and the demographic categories as predictor variables. Income and education were treated as continuous variables for these models. A similar multivariable linear regression model was estimated to examine the relationship between vaccination beliefs and vaccination intentions.

## RESULTS

### Survey Respondent Characteristics

Table 1 shows the survey respondent characteristics. All reported percentages in the text are survey weighted to represent the population of Kentucky. In terms of racial/ethnic background, 57.8% self-identified as non-Latino/a white, 21.6% as African American or black, and 18.2% as Latino/a. Close to one-third (32%) were from Appalachian counties, as defined by the Appalachian Regional Commission. Of those residing in Appalachia, 73% resided in rural counties, 17% resided in counties with RUCC codes 4–6, and the remaining 9% resided in counties with RUCC codes 1–3 (four Appalachian counties are adjacent to metropolitan areas).

Eleven percent of respondents reported having received one dose of COVID-19 vaccine, and 91% of these respondents reported intention to get the second dose (the survey did not ask about any single-dose vaccine). Vaccinated respondents were eliminated from further analyses of vaccine intention and hesitancy, resulting in a final analytic sample of 1,299 persons.

### Overall COVID-19 Vaccination Intention

Table 2 shows that, overall, 53% of non-vaccinated adult Kentuckians surveyed intended to get vaccinated against COVD-19 in the coming six months (or when a vaccine is available); 16% had not decided; and 31% felt that they would “probably not” or “definitely not” get vaccinated in the coming six months (or when a vaccine is available).

### General Vaccine Hesitancy and COVID-19 Vaccination Intention

Nineteen percent of non-vaccinated respondents reported hesitancy to take vaccines in general. Yet 64% of those reporting that they would “probably not” or “definitely not” get a COVID 19 vaccine reported that they had not previously refused or delayed another vaccination for themselves or a child, indicating independence between COVID-19 vaccine intention and hesitancy to take other vaccines.

### Associations Between Demographic Factors and COVID-19 Vaccination Intention

In multivariate regression analyses, significant associations were found between COVID-19 vaccine intention and education, race, ethnicity, income, political affiliation, rurality, and gender (Table 3). Lower intention to be vaccinated was independently associated with young adult age, lower educational attainment, black/African American race, American Indian/Alaskan Native race, lower income, Republican political affiliation, rural residence, and female gender.

Those residing in Appalachian counties had, in descriptive terms, lower vaccination intentions than those in non-Appalachian counties (3.04 versus 2.05, respectively; see Table 3). When residence in an Appalachian County was examined in a univariable model, this difference was statistically significant (*b*= −0.41, 95% CI: −0.65, −0.17). However, as can be seen in Table 3, in a multivariable model including rurality of residence, the effect for Appalachian residence is not significant.

### Associations Between COVID-19 Vaccine Beliefs and Vaccination Intention

Multivariate regression analyses also found significant associations between COVID-19 vaccine intention and vaccine attitudes and beliefs (Table 4). Lower intention to be vaccinated was independently associated with beliefs that the vaccines were not safe, were not effective, that they might cause lasting health problems, and that the COVID-19 vaccines were being pushed to make money for drug companies. A “wait-and-see” attitude (coupled with belief that the vaccines were so new) was also associated with low vaccination intention. Neither rurality nor Appalachian residence moderated the relations of beliefs and intentions (all slope tests *t*<1, *ns).*

## DISCUSSION

To our knowledge, this was only the second population-based survey of Kentucky adults’ intentions to be vaccinated against COVID-19 infection; a repeat of the first was conducted in August 2021.^17^,^18^ This study is unique in its reporting of associations of Appalachian residence in Kentucky with COVID-19 vaccination intention and beliefs. A telephone-based survey of 807 responding Kentucky adults was conducted by the Foundation for a Healthy Kentucky in February and March 2021. Despite some differences in methodology, this survey of Kentucky residents yielded results similar to ours in terms of overall vaccination intent, and the direction of the effects of demographic subsets of age, gender, political affiliation, education, and rurality.^17^ Our results add to these findings with multifactorial analyses of the associations of multiple demographic factors with COVID-19 vaccination intention. The general results of both Kentuckyspecific surveys are similar to those reported from other surveys with varying sample-frames.^3^,^8^,^9^ The Centers for Disease Control and Prevention (CDC) began assessing vaccine confidence for the COVID-19 vaccine at the national and state level in summer 2021. Kentucky’s most recent data from the Vaccine Confidence Dashboard revealed that 19.8% probably or definitely would not get vaccinated, and 9.2% were unsure or might get vaccinated.^19^ Factors associated with lack of vaccine confidence included younger age (18–49 years), rural location, income below poverty level, uninsured status and male gender.^18^

## IMPLICATIONS

Hesitancy or frank resistance to accepting vaccination against COVID-19 remain significant barriers to emergence from this pandemic, which continues to disrupt lives and economies throughout the world. In order to effectively educate and motivate unvaccinated persons to accept vaccination, health practitioners— clinicians, public health professionals, and health educators alike—need to understand the prevalence and specific correlates of COVID-19 vaccine hesitancy and resistance among the people they serve. This study identified several factors as associated with low intention to be vaccinated against COVID-19 among Kentuckians: rural residence, black/African American race, lower income, lower educational attainment, young-adult age, Republican political affiliation, and female gender. Since the survey asked respondents to simply indicate their political party *affiliation*, no conclusions can be drawn about their actual voting patterns. Beliefs that COVID-19 vaccines are unsafe and/or not effective, and mistrust in vaccine producers were also associated with low intention to be vaccinated. These findings should be used in context with the growing body of knowledge about vaccine hesitancy to inform and better tailor patient education materials, public service announcements, and health promotion campaigns about COVID-19 vaccines.

This study has two main limitations common to survey research: its cross-sectional nature and potential response biases that could not be measured. Because the survey was conducted as new vaccines were introduced, in rapidly changing policy and information environments, these limitations may be greater than for surveys done in less dynamic milieus. Nevertheless, the dual-mode methods used to collect responses (with control for mode in our analyses) and the weighted analysis techniques allowed for population-representative inferences of results. Thus, this study has the strengths of sound methodology for population-based sampling across geographic regions in Kentucky, purposeful over-sampling to support focused analyses of the effects of three strata of race and ethnicity (black/African American, white, and Latino/a), and a large sample size.

**SUMMARY BOX**

**What is already known about this topic?**

Rates of COVID-19 vaccination intention and hesitancy, plus related demographic and attitudinal correlates, have been reported for various populations, but such population-based data specific to Kentucky and to Appalachia are lacking.

**What is added by this report?**

This report of the findings of a recent population-based survey of Kentucky residents, with over-sampling for rural and African American residents, provides information specific to Kentuckians and to Appalachian and other subpopulations within Kentucky.

**What are the implications for future research?**

These findings can be used to create and study the impacts of evidence-based patient education materials, public service announcements and health promotion campaigns about COVID-19 vaccination tailored to race, ethnicity, rurality, political affiliation, and Appalachian residence.

## Figures and Tables

**Table 1:** Sample Characteristics from Survey Responses (n=1,299 Kentucky non-vaccinated adults, January–March 2021)

Demographic Variable	Percentage of Sample	
	Scaled to the KY Population	Raw, unadjusted
**Appalachian Residence**	26.2	20.9
Urban/Non-rural (RUCA 1–6)	67.9	73.7
Rural (RUA 7–9)	32.1	26.3
**Age**		
18–24	12.23	11.77
25–34	16.79	20.22
35–44	16.08	23.61
45–54	17.00	18.84
55–64	17.24	14.47
65+	20.66	11.09
**Gender**		
Male	49.24	33.24
Female	50.76	66.48
Other	0.00	0.27
**Education Level**		
Less than high school degree	5.75	6.86
High school degree / GED	31.25	28.60
Some college*	22.64	23.27
Certificate or technical degree	3.99	3.67
Associate degree, Certificate or Technical Degree	9.71	10.87
Bachelor’s degree	13.28	15.17
Master’s degree	10.46	9.56
Professional or doctoral degree (e.g., PhD, MD, JD)	2.64	2.01
**Race / Ethnicity**		
White	86.63	57.77
Black or African American	9.41	24.61
American Indian or Alaska Native	0.85	1.64
Asian or Pacific Islander	0.66	1.44
Latino/a	4.62	18.23
Middle Eastern or North African	0.12	0.27
Other	0.62	1.23
Multiracial	1.73	3.45
**Income Level**		
<$10,000	11.59	13.95
$10,000 – $14,999	9.51	8.36
$15,000 – $19,999	9.03	8.79
$20,000 – $34,999	19.23	17.51
$35,000 – $49,999	16.28	15.33
$50,000 – $74,999	15.43	15.92
$75,000 – $99,999	8.47	8.94
$100,000 – $199,000	8.67	9.45
>$200,000	1.79	1.74
**Insurance Status**		
Insurance through employer / union	33.67	36.39
Insurance purchased directly	8.28	8.22
Medicare, Medicaid, or other government source	52.15	45.65
TRICARE or other military health insurance	3.30	3.84
Veterans Affairs Insurance	3.63	2.95
Other	9.43	10.01
Uninsured	2.57	5.07
**Health Provider** (outside of mental health professionals)		
Yes	65.67	59.00
No	34.33	41.00
**Political Affiliation**		
Republican	31.35	23.96
Democrat	32.63	38.41
Independent	24.52	22.03
Other	11.5	15.59
**Employment Status**		
Employed	40.64	47.26
Self-employed	8.19	8.98
Looking for work	7.75	8.64
Unable to work due to a disability	12.17	11.04
On temporary layoff from a job	2.32	2.88
Retired	23.16	14.40
Student	4.22	5.35
Stay-at-home spouse or partner	6.34	7.96

**NOTES:**

Questions giving option of “check all that apply” have total responses >100%.

* Some college but no degree, including those currently enrolled in college.

**Table 2:** Intention to Take COVID-19 Vaccination When Available (n=1,299 Kentucky non-vaccinated adults, January–March 2021)

Measure	Definitely No/Very Unlikely	Probably Not/Unlikely	I Don’t Know	Probably Yes/Likely	Definitely Yes/Very Likely
Will you take it?	13%	14%	12%	23%	39%
How likely are you to take it?	20%	14%	10%	25%	31%
Overall Intentions (average of 2 items above)*	17%	14%	16%	25%	28%

**NOTES:**

* Rounding of mean group responses caused apparent discrepancies between rows.

**Table 3:** Mean Vaccination Intentions* and Relation of Demographic and Socioeconomic Factors to Vaccination Intentions, Multivariate Analysis (n=1,299 Kentucky non-vaccinated adults, January–March 2021)

Demographic Characteristic	Vaccination Intentions Mean (95% CI)	Slope (95% CI)
**Appalachian Residence**		−0.16 (−0.39, 0.07)
Appalachian	3.04 (2.83, 3.24)	
Non-Appalachian	3.45 (3.33, 3.57)	
***Age***		*0.16 (0.09, 0.23)*
*<25*	3.18 (2.94, 3.42)	
*25–34*	2.97 (2.75, 3.18)	
*35–44*	3.14 (2.94, 3.34)	
*45–54*	3.17 (2.93, 3.42)	
* 55–64*	3.50 (3.24, 3.77)	
* ≥65*	3.92 (3.65, 4.20)	
***Education***		*0.12 (0.06, 0.18)*
*Less than High School degree*	3.02 (2.65, 3.39)	
*High school degree or GED*	3.04 (2.86, 3.22)	
* Some college but no degree (including currently enrolled in college)*	3.32 (3.12, 3.53)	
* Certificate or technical degree*	2.81 (2.21, 3.41)	
* Associate degree*	3.18 (2.89, 3.47)	
* Bachelor’s Degree*	3.98 (3.74, 4.22)	
* Master’s Degree*	4.00 (3.63, 4.36)	
* Doctoral Degree (e.g., PhD, MD, JD)*	3.87 (3.13, 4.62)	
***Race/Ethnicity***		
White/Non-Hispanic	3.31 (3.19, 3.43)	REF
* Black/Non-Hispanic*	3.46 (3.26, 3.66)	*−0.28 (−0.54, −0.01)*
* Hispanic or Latino/a*	3.86 (3.69, 4.04)	*0.40 (0.15, 0.65)*
* American Indian/Alaskan Native*	1.49 (0.56, 2.41)	*−2.13 (−2.35, −1.9)*
Asian/Pacific Islander	3.74 (3.11, 4.37)	0.38 (−0.34, 1.09)
Middle Eastern/North African	3.16 (1.13, 5.19)	−0.81 (−3.6, 1.96)
Other	2.29 (1.22, 3.37)	−0.46 (−1.43, 0.50)
Multiracial	3.07 (2.60, 3.55)	−0.44 (−0.91, 0.02)
***Income***		*0.08 (0.02, 0.13)*
$0–9,999	2.76 (2.53, 2.99)	
$10,000–$14,999	3.06 (2.72, 3.40)	
$15,000–$19,999	3.16 (2.85, 3.46)	
$20,000–$34,999	3.33 (3.09, 3.58)	
$35,000–$49,999	3.59 (3.31, 3.87)	
$50,000–$74,999	3.58 (3.31, 3.85)	
$75,000–$99,999	3.55 (3.22, 3.87)	
$100,000–$199,999	3.69 (3.31, 4.06)	
$200,000 or more	2.86 (2.04, 3.69)	
***Political Affiliation***		
Republican	3.00 (2.79, 3.20)	REF
Independent	3.11 (2.95, 3.27)	0.21 (−0.06, 0.47)
*Democrat*	3.93 (3.79, 4.08)	*0.83 (0.57, 1.08)*
***Urban/Rural*** (RUCC Codes, higher numbers=more rural)		*−0.05 (−0.09, −0.01)*
1	3.57 (3.40, 3.73)	
2	3.43 (3.19, 3.67)	
3	3.53 (3.18, 3.87)	
4	3.04 (2.13, 3.95)	
5	3.24 (2.83, 3.65)	
6	2.96 (2.59, 3.33)	
7	3.19 (2.91, 3.46)	
8	2.48 (1.81, 3.15)	
9	2.92 (2.53, 3.31)	
***Gender***		*−0.21 (−0.42, −0.003)*
Male	3.45 (3.28, 3.62)	
Female	3.23 (3.11, 3.35)	
**Employment Status**		
Unemployed	3.09 (2.81, 3.36)	REF
Employed	3.30 (3.16, 3.45)	−0.07 (−0.39, 0.25)
Other (student, retired, etc.)	3.43 (3.26, 3.60)	0.12 (−0.19, 0.43)
**Insurance Status**		
Uninsured	3.17 (2.62, 3.72)	REF
Medicaid/Medicare	3.29 (3.14, 3.45)	0.25 (−0.55, 1.05)
Insured, other types (e.g., employer, VA)	3.31 (3.16, 3.46)	0.09 (−0.72, 0.89)

**NOTES:**

* Higher vaccination intention signified by higher value

Beliefs in *green italics* are significantly related to vaccination intentions at *p*<0.05.

**Table 4:** Relation of COVID-19 Vaccination Beliefs and Intentions, Controlling for Demographic Variables (n=1,299 Kentucky adults, January–March 2021)

Beliefs	Slope (95% CI)
*I will feel safe getting the COVID-19 vaccine. (reverse scored)*	*−0.61 (−0.74, −0.49)*
The COVID-19 vaccine might cause short term problems, like fever or discomfort.	−0.001 (−0.11, 0.11)
*The COVID-19 vaccine is being pushed to make money for drug companies.*	*−0.14 (−0.25, −0.04)*
*The COVID-19 vaccine might cause lasting health problems.*	*−0.30 (−0.44, −0.16)*
I am concerned that the COVID-19 vaccine will cost more than I can pay.	−0.07 (−0.16, 0.03)
*The COVID-19 vaccine is so new that I want to wait a while before deciding if I should get it.*	*−0.12 (−0.23, −0.01)*
*I think the COVID-19 vaccine will be effective in preventing the virus (reverse scored).*	*−0.27 (−0.39, −0.16)*
I think it would be hard to find a provider or clinic that is easy to get to.	0.11 (−0.01, 0.23)
I think it would be hard to find a provider or clinic that has the vaccine available.	0.10 (−0.01, 0.20)

**NOTES:**

A negative slope represents lower intention for vaccination.

Beliefs in *green italics* are significantly related to vaccination intentions at *p*<0.05.
